# Comprehensive mathematical modeling of age-dependent oocyte quality and quantity for predicting live birth rate

**DOI:** 10.3389/fendo.2025.1595970

**Published:** 2025-06-09

**Authors:** Toshio Sujino, Tatsuyuki Ogawa, Akira Komiya, Makiko Tajima, Yuko Takayanagi, Yurie Nako, Hayata Nakajo, Kenichiro Hiraoka, Isao Tamura, Hidetoshi Yamashita, Kiyotaka Kawai

**Affiliations:** ^1^ Department of Reproductive Medicine, Kameda IVF Clinic Makuhari, Chiba-shi, Chiba, Japan; ^2^ Department of Urology, Kameda Medical Center, Kamogawa-shi, Chiba, Japan; ^3^ Department of Obstetrics and Gynecology, Yamaguchi University School of Medicine, Ube-shi, Yamaguchi, Japan; ^4^ Research Laboratory, H.U. Group Research Institute G.K., Akiruno-shi, Tokyo, Japan

**Keywords:** ovarian aging, fertility decline, anti-Müllerian hormone, live birth rate, prediction, curve fitting, mathematical modeling

## Abstract

**Background:**

Age-related decline in fertility is widely recognized. However, a quantitative evaluation of changes in oocyte quality and quantity remains insufficient. Therefore, developing a mathematical model to quantitatively predict live birth rates affected by these changes is essential for supporting decision-making in assisted reproductive technology.

**Methods:**

In this retrospective cohort study, we developed a mathematical model to predict live birth rates based on oocyte quality and quantity using IVF treatment data from our clinic over an 8-year period. In the first stage, medically meaningful model functions were selected, and curve fitting was performed using weighted nonlinear least-squares regression to quantify age-related changes in oocyte quality and quantity. For oocyte quality, a comparative analysis was conducted on our clinical data and other large-scale datasets, modeling the live birth rate per single vitrified-warmed blastocyst transfer (SVBT) in correlation with the euploidy rate. For oocyte quantity, the distributions of anti-Müllerian hormone levels, antral follicle count, mature oocyte count, and transferable embryo count were analyzed by two-dimensional weighted nonlinear least-squares regression. In the second stage, logistic regression was applied to analyze live birth rates per SVBT and oocyte pick-up, incorporating multiple explanatory variables.

**Results:**

The adjusted R-squared values for the curve fitting results were above 0.9, indicating high fitting accuracy. In oocyte quality evaluation, all datasets showed that the values declined to half their peak by the age of 40 years. With respect to oocyte quantity, complete distribution characteristics were successfully modeled, enabling calculations at any percentile value. Logistic regression analysis incorporating blastocyst grade and culture duration as explanatory variables allowed for embryo selection based on a single indicator (i.e., the live birth rate). In the predictive model for live birth rate per oocyte pick-up, which included age, AMH levels, and number of retrieval cycles as explanatory variables, logistic regression analysis showed an AUC of 0.84 and an accuracy of 76.4%, demonstrating high predictive performance.

**Conclusion:**

Mathematical models of age-dependent oocyte quality and quantity were successfully developed. These models were integrated to construct a multi-variable predictive tool for estimating live birth rates, offering valuable insights for reproductive decision-making.

## 1 Introduction

The decline in fertility with advancing age is a well-documented phenomenon, and the trend toward later marriages poses a significant issue in industrialized countries. With the increasing awareness of oocyte aging, elective oocyte cryopreservation has emerged as one of the available options. The euploidy rate reportedly decreases with age, suggesting a decline in oocyte quality (1). The live birth rate following euploid blastocyst transfer has been reported to be 60%, and a previous study on elective oocyte cryopreservation predicted live birth rates by multiplying the euploidy rate by 0.6 (2, 3).

Oocyte quantity also declines with age. Anti-Müllerian Hormone (AMH) is a primary biomarker for assessing the ovarian reserve. AMH is strongly correlated with both follicle-stimulating hormone (FSH) and antral follicle count (AFC), which are also commonly used to evaluate ovarian reserves (4). However, AMH and AFC have become the primary methods for assessing the ovarian reserve. AMH is secreted by antral follicles and directly reflects the number of follicles in the ovaries, whereas FSH is an indirect indicator. Additionally, AMH levels remain relatively constant throughout the menstrual cycle, providing reliable results regardless of the timing of measurement (5, 6). Although AMH levels decrease with age, the distribution of AMH levels is well known to considerably vary among women of the same age. When describing age-related fluctuations in AMH levels, a common approach is to present the distribution within an age group using means or medians, along with standard deviations (SDs). However, because AMH levels do not follow a normal distribution, means with SDs alone are insufficient to fully characterize distributional properties (7). Additionally, studies predicting the optimal number of mature oocytes required for live births reported that the distribution of mature oocyte count exhibits a pattern similar to that of AMH levels (8).

The relationship between live birth rate and explanatory variables such as age and the number of mature oocytes is typically nonlinear. When a dependent variable exhibits nonlinear relationships with multiple explanatory variables, generalized additive models can be employed for analysis (9). While generalized additive models provide flexibility to capture nonlinearity and account for complex interactions among factors, they pose a challenge in independently interpreting the effects of each variable.

When functionally modeling the nonlinear relationship between an independent variable and a dependent variable, polynomial regression is frequently applied (1, 10). Although polynomial regression is highly flexible as it can fit almost any curve, it carries a risk of overfitting by capturing small-sample errors. Moreover, the optimized parameters do not provide clinically meaningful information. Therefore, selecting a medically meaningful model function, where its optimized parameters offer clinically interpretable insights, is critically important.

Building upon these considerations, we adopted a two-stage approach. At the first stage, the relationships between age and oocyte quality and quantity were mathematically modeled using theoretically meaningful functions instead of polynomial regressions. As a result, the optimized parameters provide medically meaningful insights. At the second stage, these obtained results were integrated to develop a comprehensive mathematical model for predicting live birth rates.

## 2 Materials and methods

The analyses included individuals aged 27–45 years. In this study, the following four aspects were analyzed: (i) age versus oocyte quality, which was analyzed using weighted nonlinear least-squares regression (WNLSR); (ii) age versus oocyte quantity, which was examined using two-dimensional nonlinear least-squares regression (TWNLSR); (iii) live birth rate per single vitrified-warmed blastocyst transfer (SVBT), which was investigated by logistic regression; and (iv) live birth rate per oocyte pick-up (OPU), which was analyzed using logistic regression.

Medically meaningful model functions were selected instead of using a polynomial regression of real data to analyze age-related changes in oocyte quality and quantity. Additionally, the WNLSR or TWNLSR was employed to quantify age-related changes in oocyte quality and quantity. The term “weighted” refers to a technique used to minimize the impact of small-sample errors, particularly in age groups with fewer data points, such as younger and older individuals. In this study, weights were applied based on sample size to ensure robust curve fitting. A detailed explanation of WNLSR, along with a comparison to other regression methods and illustrative examples, is provided in **Supplementary Document S1**.

Prior to conducting logistic regression analysis, we conducted a preliminary investigation into the relationships between live birth rate and various factors. Based on this analysis, we selected the three most influential predictors as explanatory variables for the models. Logistic regression analysis of live birth rate was then conducted in the following three steps: (i) curve fitting using WNLSR: the relationship between each explanatory variable and the live birth rate was modeled using WNLSR; (ii) scaling of explanatory variables: the explanatory variables were appropriately scaled to ensure a linear relationship between the logit function and the WNLSR results; and (iii) execution of logistic regression analysis: logistic regression analysis was performed using the scaled explanatory variables.

The curve-fitting results obtained from the WNLSR were evaluated based on adjusted R-squared (R²) values to assess the fitting accuracy, and the optimized parameters were assessed using 95% confidence intervals (95% CIs). The predictive accuracy of logistic regression analysis was determined using the area under the curve (AUC) and accuracy. Regression coefficients were evaluated using 95% CIs, standard errors, adjusted odds ratios (aOR), and p-values. All analyses were conducted using Python version 3.12.2 (Python Software Foundation, Wilmington, DE, USA).

### 2.1 Age versus oocyte quality: WNLSR

For oocyte quality, we mathematically modeled the live birth rate per SVBT, which correlated with the euploidy rate. The following three datasets with significantly different sample sizes were compared to validate the reliability of the WNLSR results:

- Japan Society of Obstetrics and Gynecology (JSOG) data: nationwide data from the JSOG in 2017 (N = 125,674) (11).
- Preimplantation genetic testing for aneuploidy (PGT-A) data: data obtained by multiplying the euploidy rate by a live birth rate of 60% (N = 14,614) (1–3)
- Kameda data: data from 2,073 patients who underwent embryo transfer at our clinic from May 2016 to June 2024 (N = 4,069)

The patient backgrounds for our clinical dataset are provided in **Supplementary Table S1**. As of 2017, PGT-A had not yet been approved for clinical use in Japan; therefore, the JSOG dataset does not include blastocyst transfer cycles involving PGT-A. In addition, 23 blastocyst transfer cycles involving PGT-A were excluded from Kameda dataset. For the JSOG dataset, which had the largest sample size, the data clearly followed a logistic curve. Therefore, the WNLSR was applied to all three datasets based on a logistic equation. The parameters optimized in this curve- fitting process were as follows: (i) maximum live birth rate, defined as the highest achievable live birth rate; (ii) half-peak age, defined as the age at which the live birth rate decreased to half of its maximum value; and (iii) decline width, which determined the shape of the curve. By optimizing these parameters, the age at which the live birth rate declined to 90%, 75%, 50%, 25%, and 10% of its maximum value could be possibly calculated. Additionally, while the PGT-A dataset was generated by multiplying the euploidy rate by a fixed live birth rate of 60%, removing this multiplication yielded pure euploidy rate data in which the maximum live birth rate corresponded to the highest euploidy rate. Therefore, as a secondary analysis, we applied the WNLSR to the euploidy rate. The mathematical equations, detailed parameter descriptions, and figures are provided in **Supplementary Document S2**.

### 2.2 Age versus oocyte quantity: TWNLSR

For oocyte quantity, we mathematically modeled the distributions of AMH levels, antral follicle count (AFC), mature oocyte count, and transferable embryo count per OPU. AMH and AFC served as biomarkers for predicting the number of mature oocytes and transferable embryos, and their correlation coefficients were 0.7 or higher. Given this strong correlation, the potential for multicollinearity was considered high, and each factor was therefore analyzed separately. Henceforth, these four factors are collectively referred to as “quantifying factors” in this study.

The mean and mode, when modeled as functions of age, can also be approximated using a logistic equation. However, it is necessary to mathematically model the entire distribution within each age group. For distribution modeling, histograms were generated for each age group that exhibited a gamma distribution pattern. Based on this observation, the gamma distribution function was used as the model function, and curve fitting was performed using the TWNLSR.

The two parameters of the gamma distribution are related to the mean and mode. These two parameters were determined by optimizing the mean and mode as functions of age using a logistic equation, allowing the entire distribution to be represented mathematically. Additionally, because the cumulative gamma distribution enables the calculation of arbitrary percentile values, we computed not only the mean, mode, and median but also the 5th, 25th, 75th, and 95th percentile values. The mathematical equations, detailed parameter descriptions, and corresponding figures are provided in **Supplementary Document S3**.

The distribution of AMH levels was analyzed using 5,638 measurements conducted at our clinic on 4,972 individuals from May 2016 to December 2024. All AMH measurements were performed using cobas e 411 analyzer with the Elecsys^®^ AMH assay (Roche Diagnostics, Basel, Switzerland). To quantify factors other than AMH, data of 2,698 first oocyte retrieval cycles at our clinic from May 2016 to October 2024 were used. Patient backgrounds and ovarian stimulation protocols for first oocyte retrieval are presented in **Supplementary Table S2**. Oocyte retrieval cycles for fertility preservation and elective oocyte cryopreservation were excluded from analysis.

### 2.3 Live birth rate per SVBT: logistic regression

To predict live birth rates, we performed a logistic regression analysis using three explanatory variables related to blastocyst quality—namely, age, blastocyst grade, and culture duration. The blastocyst grade, based on Gardner’s grading system, was categorized into four groups: excellent (AA), good (AB or BA), average (BB), and fair (BC or CB). Blastocysts graded as CA (15 cycles) and CC (5 cycles) were excluded due to extremely small sample sizes and the absence of discernible outcome trends. Although the number of AC blastocyst cycles was also relatively small (54 cycles), their clinical outcomes were similar to those of BB blastocysts; therefore, they were grouped together with BB blastocysts. As the relationship between blastocyst grade and live birth rate exhibited an approximately linear trend, we applied the WNLSR using a linear function. The culture duration was a binary variable (day 5 or day 6); therefore, no special preprocessing was required.

### 2.4 Live birth rate per OPU: logistic regression

To analyze the impact of oocyte quantity, we conducted a logistic regression analysis on live birth rate per OPU using three explanatory variables—namely, age, quantifying factors, and number of retrieval cycles. As there were four types of quantifying factors, the logistic regression analysis was conducted four times, once for each factor. By including the number of retrieval cycles as an explanatory variable, we were also able to calculate the cumulative live birth rate according to the number of retrieval cycles.

As a preprocessing step, the relationship between age and live birth rate was modeled using the WNLSR with a logistic function, as in the SVBT analysis. The relationships between the quantifying factors and live birth rates exhibited the same curve pattern, which followed the trend for cumulative probability function. Therefore, the WNLSR was applied using the cumulative probability formula. The relationship between the number of retrieval cycles and live birth rate followed an exponentially decreasing curve. Consequently, the WNLSR was applied using an exponentially decreasing function. The mathematical equations, detailed parameter descriptions, and corresponding figures are provided in **Supplementary Document S4**.

The study population was defined as the data collected up to the period when the outcomes of all oocyte retrieval cycles were confirmed. Specifically, we used 4,181 oocyte retrieval cycles from 2,059 individuals, performed between May 2016 and June 2023. This dataset included all oocyte retrieval cycles, including those in which no mature oocytes or transferable embryos were generated. Additionally, in cases where transferable embryos were obtained, the dataset included cycles in which all good-quality blastocysts (BB or higher) were used for embryo transfer or cycles in which live birth (or ongoing pregnancy) was confirmed. In cases where patients had not completed the transfer of all available good-quality blastocysts, the following cycles were excluded due to the inability to determine clinical outcomes: 14 cycles were discontinued due to spontaneous pregnancy, and 54 cycles were discontinued for patient-related reasons such as divorce, relocation, or health issues.

## 3 Results

### 3.1 Age versus oocyte quality

**Figure 1:**
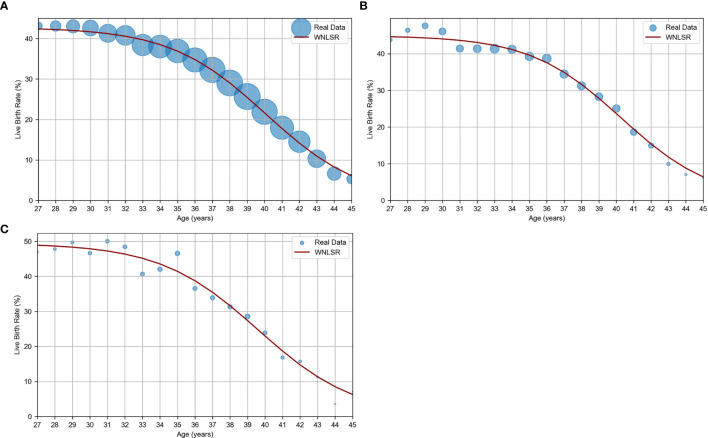
WNLSR results for age versus oocyte quality. **(A)** JSOG data: live birth rate estimated from SVBT pregnancy and miscarriage rates in Japan in 2017 (11). **(B)** PGT-A data: live birth rate estimated based on euploidy rates (1) and a constant euploid live birth rate (2, 3). **(C)** Kameda data: live birth rate per SVBT at Kameda IVF Clinic Makuhari from May 2016 to June 2024. The dot size of actual data points is proportional to the sample size, allowing for the visualization of differences in sample sizes.

The results of applying WNLSR to the three datasets are presented in **Table 1** and **Figure 1**. The dot size of actual data points in **Figure 1** is proportional to the sample size, allowing for the visualization of differences in sample sizes. The accuracy of the WNLSR was evaluated using R², which was above 0.9, indicating high goodness-of-fit. However, R² tended to increase with larger sample sizes, and the 95% CIs of each parameter tended to decrease as the sample sizes increased. For all three datasets, the rounded half-peak age and decline width were 40 and 3 years, respectively. The ages at which the maximum live birth rate decreased to 90%, 75%, 50%, 25%, and 10% were 34, 37, 40, 43, and 46 years, respectively. Notably, in the PGT-A dataset, when analyzing the pure euploidy rate without multiplying it by the assumed live birth rate of 60%, the half-peak age and decline width were exactly the same as in the standard analysis. Detailed data for the JSOG, PGT-A, and Kameda datasets are presented in **Supplementary Table S3**.

**Table 1:** WNLSR results for oocyte quality across three datasets.

Parameter	Symbol	Unit	Data sets			Euploidy
			JSOG	PGT-A	Kameda	
Sample size	N	cycles*	125,674	14,614	4,069	14,614
Adjusted R-squared	R^2^	–	0.997	0.979	0.947	0.979
Maximum value**	y_0_	%	42.7 (41.9–43.6)	44.9 (43.2–46.7)	49.4 (45.7–53.1)	74.9 (72.0–77.8)
Half-peak age	x_0_	years	40.1 (39.9–40.2)	40.3 (39.9–40.7)	39.6 (39.0–40.3)	40.3 (39.9–40.7)
Decline width	ω	years	3.02 (2.82–3.22)	2.89 (2.38–3.40)	3.07 (2.19–3.96)	2.89 (2.38–3.40)

Values within parentheses indicate 95% CIs. The “Euploidy” column results were based on the WNLSR without applying the fixed live birth rate of 60% to the PGT-A data.

*The unit for the “PGT-A” and “Euploidy” columns is “tests”.

**The “Euploidy” column represents the maximum euploidy rate, whereas the other columns show live birth rates.

### 3.2 Age versus oocyte quantity

**Figure 2:**
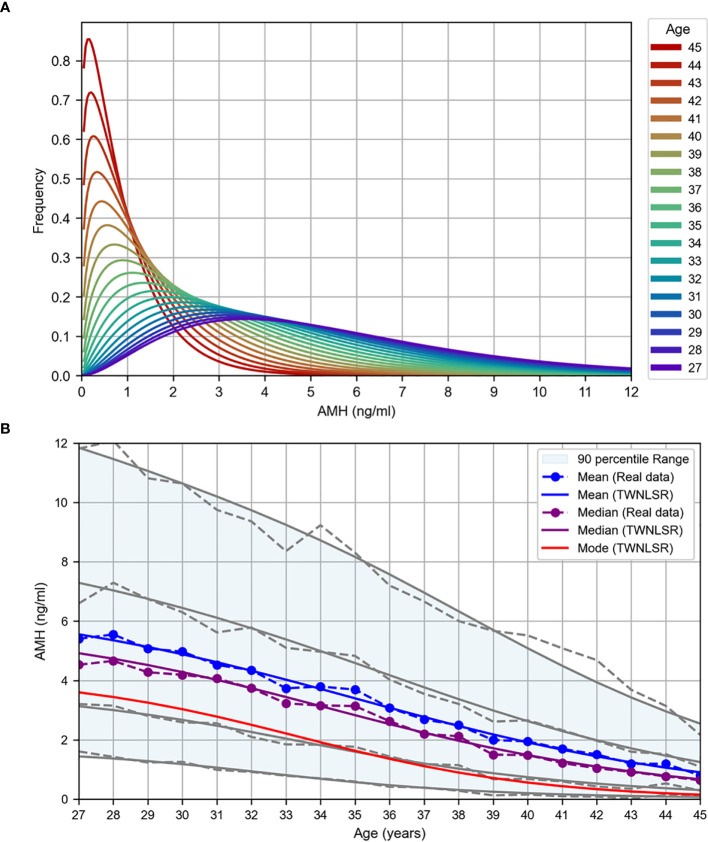
TWNLSR results for age versus oocyte quality. **(A)** AMH distribution across different ages. **(B)** Age-related changes in AMH levels measured at Kameda IVF Clinic Makuhari from May 2016 to December 2024. The mean (blue), median (purple), and mode (red) are displayed. The gray lines represent the percentile values in descending order: 95th, 75th, 25th, and 5th percentiles.

The results of the TWNLSR analysis for the quantifying factors are presented in **Table 2**. The AMH distribution graph for each age group is shown in **Figure 2A**, and age-dependent changes in AMH levels are illustrated in **Figure 2B**. In **Figure 2B**, the mean, median, and mode are displayed, along with the 5th, 25th, 75th, and 95th percentiles. The dotted lines represent the actual data. Despite optimizing only the mean and mode, the analysis results closely followed all percentile values, suggesting that curve fitting with the TWNLSR effectively captured the characteristics of the data distribution. Detailed data for each quantifying factor are shown in **Supplementary Table S4**.

**Table 2:** TWNLSR results for age-related changes in oocyte quantity across four quantifying factors.

			Quantifying factors							
			AMH		AFC		Mature oocyte count		Transferable embryo count	
Parameter	Symbol	Unit	Mean	Mode	Mean	Mode	Mean	Mode	Mean	Mode
Maximum value	y0	count*	6.5 (6.0–7.0)	3.8 (3.5–4.2)	22.6 (21.3–24.0)	18.0 (15.8–20.1)	14.5 (13.9–15.1)	9.8 (8.9–10.6)	5.3 (5.1–5.5)	2.8 (2.5–3.1)
Half-peak age	x0	years	35.5 (34.5–36.5)	34.3 (33.6–35.1)	35.7 (34.8–36.6)	34.8 (33.2–36.4)	38.6 (38.0–39.2)	36.7 (35.8–37.7)	38.7 (38.3–39.2)	37.8 (36.6–38.9)
Decline width	ω	years	5.7 (5.3–6.1)	3.4 (3.0–3.8)	7.2 (6.6–7.7)	6.3 (5.3–7.2)	5.9 (5.4–6.4)	4.1 (3.3–4.9)	4.5 (4.0–4.9)	3.1 (2.1–4.2)
Adjusted R-squared	R^2^	–	0.950		0.970		0.972		0.972	

Values within parentheses indicate 95% CIs.

*The unit for AMH levels is “ng/mL,” whereas other quantifying factors are measured in counts.

### 3.3 Live birth rate per SVBT: logistic regression

**Figure 3:**
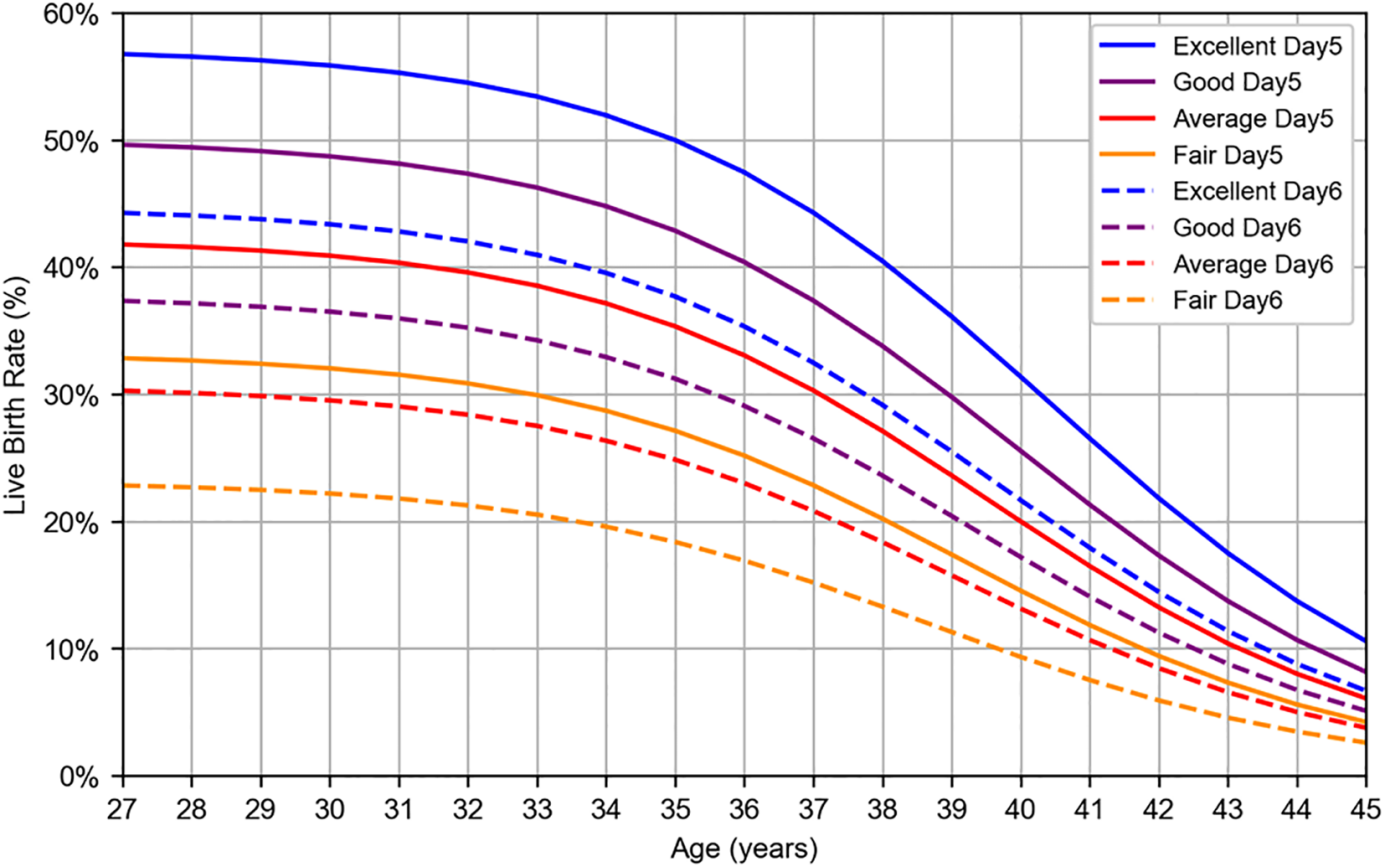
Logistic regression result for live birth rate per SVBT. Live birth rates were estimated using logistic regression analysis based on patient data collected at Kameda IVF Clinic Makuhari from May 2016 to June 2024. Blastocysts were categorized according to Gardner’s grading system: excellent (AA), good (AB, BA), average (BB), and fair (BC, CB). Solid lines represent day 5 blastocysts, whereas dashed lines represent day 6 blastocysts.

The logistic regression analysis yielded the following logit function: logit(p) = 4.552 – 0.129 × Age_scaled – 0.328 × Grade_scaled – 0.502 × Culture_duration, where logit(p) = ln(p/(1 – p)), and p represents the probability of live birth. The results of the logistic regression analysis with age, blastocyst grade, and culture duration as the explanatory variables are presented in **Table 3**, and a graph illustrating the live birth rate as a function of age is shown in **Figure 3**. The AUC and accuracy were 0.665 and 0.601, respectively, which were not particularly high. However, the analysis quantitatively revealed that as the blastocyst grade decreased, the live birth rate declined. Additionally, as the culture duration increased by one day, the live birth rate also decreased. This allowed for the selection of embryos for transfer based on a single metric (i.e., the live birth rate), even when considering both blastocyst grade and culture duration. The results of WNLSR preprocessing and the aORs obtained from logistic regression analysis are presented in **Supplementary Table S5**, and the data table of **Figure 3** is presented in **Supplementary Table S6**.

**Table 3:** Logistic regression results for live birth rate per SVBT.

Variables	Coefficient	SE	aOR	*p*-value
Age (scaled)	-0.129 (-0.150, -0.108)	0.011	0.879 (0.861, 0.898)	<0.001
Grade (scaled)	-0.328 (-0.400, -0.255)	0.037	0.720 (0.670, 0.775)	<0.001
Culture duration	-0.502 (-0.735, -0.269)	0.119	0.605 (0.479, 0.764)	<0.001

Values within parentheses indicate 95% CIs.

Model performance metrics: AUC = 0.665, optimal threshold = 0.365, accuracy = 0.601.

SE, standard error; aOR, adjusted odds ratio.

### 3.4 Live birth rate per OPU: logistic regression

**Figure 4:**
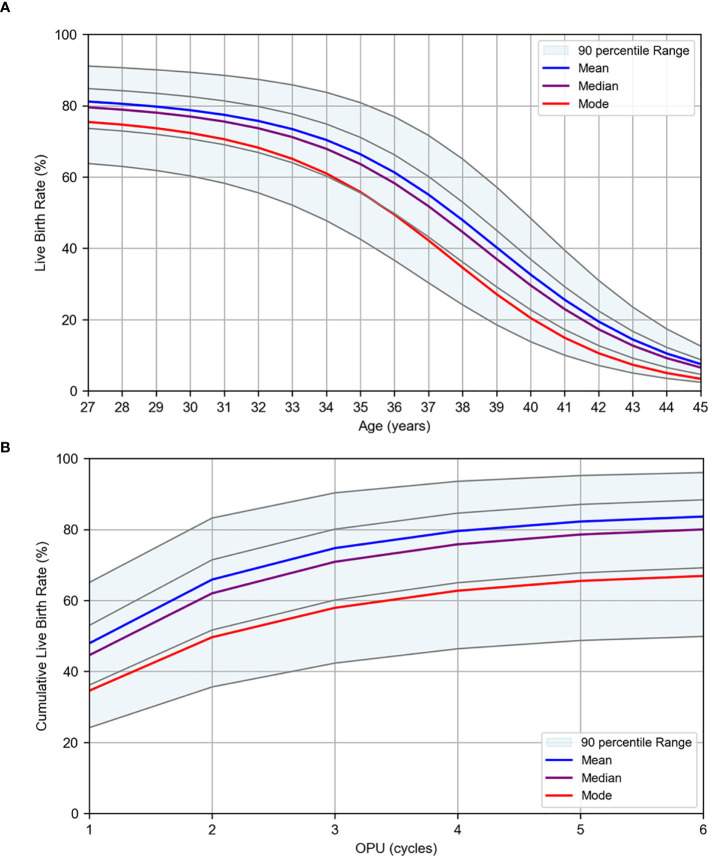
Logistic regression results for live birth rates per OPU. Live birth rates were estimated using logistic regression analysis based on patient data collected at Kameda IVF Clinic Makuhari from May 2016 to June 2023. **(A)** Live birth rate by age for first OPU cycles. **(B)** Cumulative live birth rate by OPU cycle at the average age of 38 years. The plots are stratified by AMH levels. The mean (blue), median (purple), and mode (red) are displayed. The gray lines represent the percentile values in descending order: 95th, 75th, 25th, and 5th percentiles.

The logistic regression analyses were performed separately for the following four quantifying factors, yielding distinct logit functions:

- logit(p) = 4.922 – 0.158 × Age_scaled + 0.286 × AMH_scaled – 0.185 × OPUcycle_scaled,
- logit(p) = 2.683 – 0.159 × Age_scaled + 0.211 × AFC_scaled – 0.200 × OPUcycle_scaled,
- logit(p) = 1.646 – 0.164 × Age_scaled + 0.369 × Mature_oocyte_count_scaled – 0.145 × OPUcycle_scaled,
- logit(p) = – 1.935 – 0.164 × Age_scaled + 1.361 × Transferable_embryo_count_scaled – 0.054 × OPUcycle_scaled,

where logit(p) = ln(p/(1 – p)), and p represents the probability of live birth. The results of the logistic regression analysis using age, quantifying factors, and number of OPU cycles as the explanatory variables are presented in **Table 4**. All models demonstrated high AUC and accuracy, with a tendency toward higher evaluation values for the mature oocyte count and transferable embryo count, which are directly linked to embryo transfer. This is considered a reasonable outcome. A graph illustrating the live birth rate stratified by AMH levels in the first oocyte retrieval cycle is shown in **Figure 4A**, and cumulative live birth rates stratified by AMH levels at an average maternal age of 38 years are presented in **Figure 4B**. In **Figure 4**, the mean, median, and mode are displayed along with the percentile values for each age group, which were calculated for age versus oocyte quantity. The results of WNLSR preprocessing for each quantifying factor and the aORs obtained from logistic regression analysis are provided in **Supplementary Table S7**. The calculated data for each quantifying factor in **Figures 4A, B** are presented in **Supplementary Tables S8** and **S9**, respectively.

**Table 4:** Logistic regression results for live birth rate per OPU for four quantifying factors.

Variables	Coefficient	SE	aOR	*p*-value
AMH (scaled)	0.286 (0.245, 0.328)	0.021	1.331 (1.277, 1.388)	<0.001
Age (scaled)	-0.158 (-0.177, -0.139)	0.010	0.854 (0.838, 0.870)	<0.001
OPU cycles (scaled)	-0.185 (-0.241, -0.130)	0.028	0.831 (0.786, 0.878)	<0.001
AFC (scaled)	0.211 (0.180, 0.241)	0.016	1.234 (1.197, 1.273)	<0.001
Age (scaled)	-0.159 (-0.178, -0.139)	0.010	0.853 (0.837, 0.870)	<0.001
OPU cycles (scaled)	-0.200 (-0.256, -0.144)	0.029	0.819 (0.774, 0.866)	<0.001
Mature oocyte count (scaled)	0.369 (0.330, 0.408)	0.020	1.446 (1.391, 1.504)	<0.001
Age (scaled)	-0.164 (-0.184, -0.144)	0.010	0.849 (0.832, 0.866)	<0.001
OPU cycles (scaled)	-0.145 (-0.204, -0.086)	0.030	0.865 (0.816, 0.918)	<0.001
Transferable embryo count (scaled)	1.361 (1.217, 1.505)	0.074	3.900 (3.376, 4.506)	<0.001
Age (scaled)	-0.164 (-0.185, -0.142)	0.011	0.849 (0.831, 0.868)	<0.001
OPU cycles (scaled)	-0.054 (-0.117, 0.008)	0.032	0.947 (0.890, 1.008)	0.086

Values within parentheses indicate 95% CIs.

Model performance metrics: AMH: AUC = 0.838, optimal threshold = 0.376, accuracy = 0.765. AFC: AUC = 0.839, optimal threshold = 0.364, accuracy = 0.763. Mature oocyte count: AUC = 0.865, optimal threshold = 0.327, accuracy = 0.772. Transferable embryo count: AUC = 0.908, optimal threshold = 0.411, accuracy = 0.817.

SE, standard error; aOR, adjusted odds ratio.

## 4 Discussion

### 4.1 Age versus oocyte quality

The WNLSR model function used in this study is not merely a method for functionally approximating data or a pre-processing step for logistic regression analysis. An essential aspect of this approach is that the model function itself carries medical significance, and the optimized parameters provide valuable clinical insights. The logistic equation adopted in this section was originally proposed by Verhulst in the 19th century as a mathematical model of population growth incorporating external constraints (12). By reversing the sign of the equation, it can also be applied to declining trends, such as decreases in fertility or reproductive potential. The first parameter of the logistic equation reflects the external factors, suggesting that the maximum live birth rate may improve with future advancements in medical technology. In contrast, half-peak age and decline width are intrinsic parameters defined by the logistic equation and can be regarded as “intrinsic parameters of oocyte quality.” Importantly, these intrinsic parameters remained unchanged irrespective of whether the analysis was based on the live birth rate or euploidy rate, as presented in **Table 1**. This finding underscores the importance of selecting medically meaningful model functions, as the optimized parameters of polynomial regression fail to yield clinically meaningful insights. Therefore, regardless of the maximum value, oocyte quality was calculated to have declined to 90%, 75%, 50%, 25%, and 10% of its peak at approximately 34, 37, 40, 43, and 46 years of age, respectively. These indicators may serve as a decision-making reference for patients, supporting their choice regarding the appropriate age for elective oocyte cryopreservation.

### 4.2 Age versus oocyte quantity

For oocyte quantity, this study extended beyond the mathematical modeling of mean and median values by functionally modeling the entire age-specific distribution. This approach allows for a comprehensive mathematical representation of the quantifying factors and captures their overall characteristics within a unified model. By adopting this method, we quantitatively demonstrated that, even within the same age group, the quantifying factors exhibited a wide distribution. Again, the optimized parameters of polynomial regression fail to reveal the relationship between the mean and mode, making it impossible to fully characterize the distributions. These findings contribute to improving the accuracy of patient counseling. Furthermore, previous studies have reported that AMH values can vary owing to measurement errors associated with assay methods and differences in ethnicity (13, 14). The dataset used in this study consists primarily of AMH values obtained from Japanese women. Conducting analyses similar to those in this study, while standardizing assay methods and ethnic backgrounds, may contribute to the standardization of AMH values. Clinically, the percentile values of AMH and AFC calculated in this study enabled the prediction of the number of mature oocytes and the number of transferable embryos per oocyte retrieval cycle. Additionally, if the percentile values of the mature oocyte count or transferable embryo count significantly deviate from those of AMH or AFC, further investigations focusing on specific factors such as ovarian stimulation protocols and embryo culture techniques should be conducted.

### 4.3 Live birth rate per SVBT: logistic regression

When PGT-A is not performed, blastocyst grade and culture duration are commonly used as indicators for embryo selection. However, considering both factors simultaneously can sometimes make it difficult to determine which blastocysts should be prioritized for transfer. Through logistic regression analysis, we integrated these factors, enabling embryo selection based on a single indicator—the live birth rate.

### 4.4 Live birth rate per OPU: logistic regression

Logistic regression analyses were conducted separately for each of the four quantifying factors: AMH, AFC, mature oocyte count, and transferable embryo count. This approach was adopted because these factors not only exhibited high correlations with each other but also reflected different stages within the assisted reproductive treatment process. Furthermore, each factor can be individually referenced at an appropriate stage of the treatment process. In terms of the temporal sequence, AMH is considered the next most relevant factor after age, as it can be measured solely through a blood test. The results of this study indicated that the live birth rate could be predicted by incorporating AMH levels alone (AUC: 0.84, accuracy: 76.7%), highlighting its significant clinical value. Additionally, cumulative live birth rate analysis quantitatively demonstrated that even among patients of the same age, those with lower AMH levels might require multiple oocyte retrieval cycles to achieve a live birth. This finding allows patients with lower AMH levels to better understand their treatment strategies and enables medical professionals to develop more precise and individualized treatment plans.

### 4.5 Limitations and considerations

The logistic regression results reflect the statistical effects of selected explanatory variables, while other biological or clinical factors are averaged out. As this analysis is based on retrospective data from a single institution, the predictive outcomes represent general trends and do not guarantee individual treatment outcomes.

A key limitation of this study lies in the use of data from a single institution, resulting in a relatively limited sample size. However, for age versus oocyte quality, we validated the analytical method by examining three datasets with different sample sizes. The results demonstrated that as the sample sizes increased, the R² value improved, and the 95% CIs narrowed, indicating a convergence trend toward the model function. Despite the limited sample size at our institution, the half-peak age and decline width did not show any significant discrepancies. Therefore, as long as a similar sample size is secured, analyses with comparable accuracy can be performed, even if the data are derived from a single institution.

However, a larger sample size is always preferable, as an increased number of samples allows for a more definitive selection of the model function and is expected to enhance the analytical accuracy. In reproductive medicine articles, some studies explicitly stated their sample sizes in their titles. Indeed, datasets with sufficiently large sample sizes are particularly well-suited for mathematical model analysis, similar to that conducted in this study. The objective of this study is not only to present numerical results, but also to propose a versatile analytical method. Therefore, this analytical approach should be applied in multicenter collaborative studies where sufficient sample sizes can be obtained for further validation.

### 4.6 Wider implications and future planning

One of the main strengths of this study lies in the selection of medically meaningful model functions, which enabled the optimized parameters to provide novel clinical insights. In particular, whereas no complete mathematical model had previously been established for the distribution of oocyte quantity, we successfully developed a unified mathematical model by adopting the gamma distribution to represent both age-dependent changes and within-age distributional characteristics. As a result, it became possible to predict live birth probability with reasonable accuracy using only basic information such as age and AMH levels.

Furthermore, logistic regression allows for the flexible inclusion of additional variables, making it possible to expand upon the current model by incorporating new factors of interest. By treating the explanatory variables used in this study as confounders, the independent effects of maternal factors or emerging medical technologies can be appropriately evaluated. In subsequent research, we also plan to incorporate additional variables known to influence live birth outcomes, in order to further improve predictive accuracy.

In addition, the age-dependent changes in oocyte quality and quantity identified in this study may serve as a decision-support tool for patients, aiding in individualized reproductive planning. Our findings not only provide essential information for patients considering future childbearing, but also serve as valuable data for governments and corporations to support work-life balance for individuals planning their families. Ultimately, we aim to evaluate the utility of this model in clinical practice, particularly in facilitating shared decision-making between patients and healthcare providers.

## Data Availability

The original contributions presented in the study are included in the article/**Supplementary Material**. Further inquiries can be directed to the corresponding authors.
