# Supplementary material for: Comprehensive mathematical modeling of age-dependent oocyte quality and quantity for predicting live birth rate
Source: Front Endocrinol (Lausanne). 2025 Jun 9;16:1595970. doi: 10.3389/fendo.2025.1595970 (PMC12183067; doi:10.3389/fendo.2025.1595970)
Supplement: Supplementary file 1 [file DataSheet1.zip › Supplementary Materials/Supplementary Documents/Supplementary_document_1.docx]

Supplementary Document 1. Explanation and example of weighted nonlinear least-squares regression (WNLSR)

When developing predictive tools for clinical pregnancy rate or live birth rate, it is generally inappropriate to use raw data directly, especially when considering the potential influence of small-sample bias. Therefore, it is necessary to approximate the raw data using some type of mathematical function. In any approximation method, the goal is to iteratively adjust the parameters of the function so that the residual sum of squares (RSS)—that is, the squared difference between the observed values and the predicted values—is minimized. The RSS is defined as shown in Equation (1).

$$\begin{aligned} \boldsymbol{RSS=}\sum_{\boldsymbol{i=1}}^{\boldsymbol{n}} \left( \boldsymbol{Y}_{\boldsymbol{i}}\boldsymbol{-y}\left( \boldsymbol{x}_{\boldsymbol{i}} \right) \right)^{\boldsymbol{2}}\boldsymbol{\#}\left( \boldsymbol{1} \right) \end{aligned}$$

Here, *Y_i_* represents the observed value of the i-th data point, and *y(x_i_)* denotes the predicted value generated by the function for the same *x_i_*.

1. **Linear regression**

The simplest approximation method is linear regression, which allows the user to assess only the overall direction of the relationship between the explanatory and outcome variables—whether it is increasing or decreasing—based on the raw data (scatter plot). This linear regression function is built into most standard spreadsheet software and can be easily applied. In the case of linear regression, the function *y(x_i_)* in Equation (1) is defined as shown in Equation (2).

$$\begin{aligned} \boldsymbol{y}\left( \boldsymbol{x}_{\boldsymbol{i}} \right)\boldsymbol{=a+b\cdot}\boldsymbol{x}_{\boldsymbol{i}}\boldsymbol{\#}\left( \boldsymbol{2} \right) \end{aligned}$$

Here, *a* represents the intercept and *b* represents the slope. Linear regression is a method in which the parameters *a* and *b* are iteratively adjusted so that the RSS is minimized. An example of linear regression using our clinic's AMH data is shown in Figure 1.


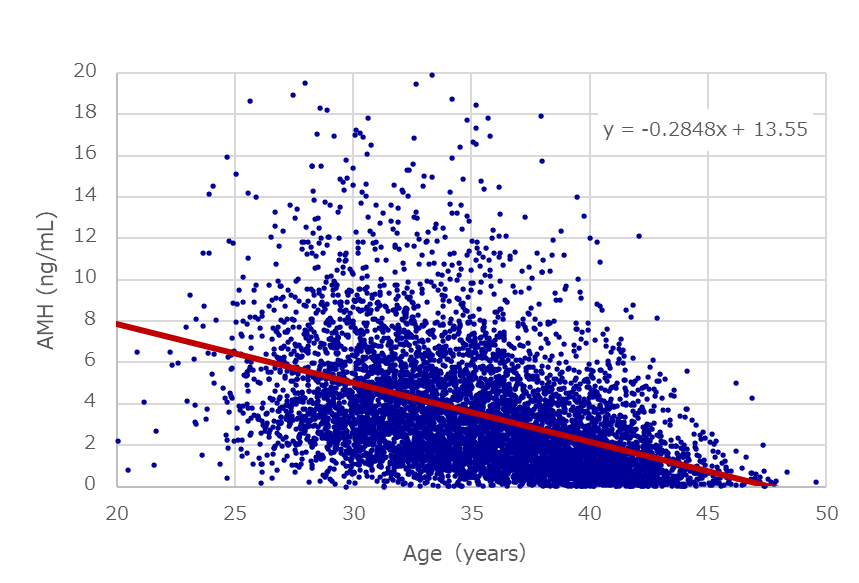


Figure 1. Linear regression of AMH as a function of female age

The blue dots represent the observed values, while the red solid line represents the linear regression line. As indicated by the regression equation shown in the upper right of the graph, the spreadsheet software calculates *a* = 13.55 and *b* = -0.2848, suggesting that AMH levels tend to decrease with increasing age. However, in reproductive medicine, data do not always follow a linear relationship and are often nonlinear in nature. Therefore, to better capture the distribution of the data, it is necessary to employ more flexible approximation methods such as polynomial regression.

1. **Polynomial regression**

Similar to linear regression, polynomial regression is also available in most standard spreadsheet software and typically supports polynomial terms up to the sixth degree. In polynomial regression, the function *y(x_i_)* in Equation (1) is defined as shown in Equation (3).

$$\begin{aligned} \boldsymbol{y}\left( \boldsymbol{x}_{\boldsymbol{i}} \right)\boldsymbol{=a+b\cdot}\boldsymbol{x}_{\boldsymbol{i}}\boldsymbol{+c\cdot}{\boldsymbol{x}_{\boldsymbol{i}}}^{\boldsymbol{2}}\boldsymbol{+d\cdot}{\boldsymbol{x}_{\boldsymbol{i}}}^{\boldsymbol{3}}\boldsymbol{+e\cdot}{\boldsymbol{x}_{\boldsymbol{i}}}^{\boldsymbol{4}}\boldsymbol{+f\cdot}{\boldsymbol{x}_{\boldsymbol{i}}}^{\boldsymbol{5}}\boldsymbol{+g\cdot}{\boldsymbol{x}_{\boldsymbol{i}}}^{\boldsymbol{6}}\boldsymbol{\#}\left( \boldsymbol{3} \right) \end{aligned}$$

In polynomial regression, it is theoretically possible to add polynomial terms of arbitrarily high degree. However, in practice, higher-degree terms tend to become excessively large, often leading to divergence and resulting in an unstable approximation. For example, in sixth-degree polynomial regression, the function defined in Equation (3) includes seven parameters—namely, *a*, *b*, *c*, *d*, *e*, *f*, and *g*—which are iteratively adjusted so that the RSS is minimized. Franasiak et al. superimposed a fifth-degree polynomial regression curve onto observed data to approximate age-specific aneuploidy rates (Refer to Reference 1). Using the euploidy rate data reported by Franasiak et al. for women aged 25 to 46 years, we performed a fifth-degree polynomial regression using standard spreadsheet software. The resulting curve is shown in Figure 2.


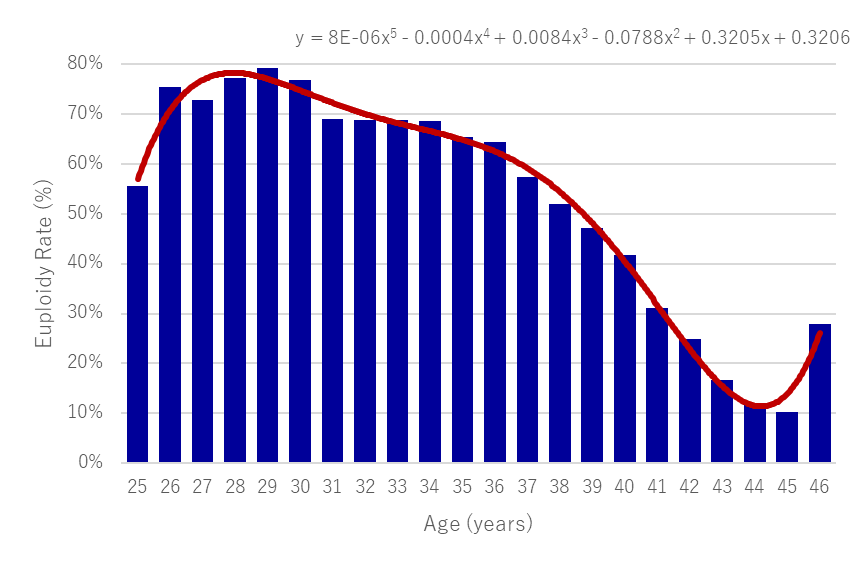


Figure 2. Fifth-order polynomial regression of euploidy rate as a function of female age

The blue bars represent the observed values, while the red solid line represents the polynomial regression curve. Adding higher-degree terms allows the fitted curve to more closely follow the actual data. However, in age groups with smaller sample sizes, small-sample variation can become more pronounced. For example, as shown in Figure 2, the euploidy rate at age 46 appears higher than at age 42. The result is unlikely to be medically meaningful and is more reasonably interpreted as random variation due to limited sample size. The fifth-degree polynomial regression shown in Figure 2 also follows such small-sample fluctuations, a phenomenon known as overfitting. Furthermore, the parameters obtained through polynomial regression often lack a direct relationship to the units of the explanatory or outcome variables, and thus typically provide little medical interpretability. Therefore, it is worth considering nonlinear regression methods that employ model functions with parameters that can be meaningfully interpreted in a medical context.

1. **Nonlinear least-squares regression**

Nonlinear least-squares regression using a model function is not commonly supported by standard spreadsheet software and often requires more advanced statistical analysis tools. Here, we consider an example in which a logistic function is used as the model function for approximation. In this case, the function *y(x_i_)* in Equation (1) is defined as shown in Equation (4).

$$\begin{aligned} \boldsymbol{y}\left( \boldsymbol{x}_{\boldsymbol{i}} \right)\boldsymbol{=}\frac{\boldsymbol{y}_{\boldsymbol{0}}}{\boldsymbol{1+}\mathbf{exp}\left( \frac{{\boldsymbol{x}_{\boldsymbol{i}}\boldsymbol{-x}}_{\boldsymbol{0}}}{\boldsymbol{\omega}_{\boldsymbol{0}}} \right)}\boldsymbol{\#}\left( \boldsymbol{4} \right) \end{aligned}$$

Here, *y₀* shares the same unit as the outcome variable. If *y* represents the live birth rate, then *y₀* corresponds to the maximum live birth rate; if *y* represents the euploidy rate, then *y₀* corresponds to the maximum euploidy rate. *x₀* shares the same unit as the explanatory variable. If *x* denotes age, then *x₀* indicates the age at which the outcome reaches half of its maximum value, that is, the half-life age. *ω₀* also shares the same unit as the explanatory variable, and if *x* denotes age, then *ω₀* represents the decay width, or the age interval over which the value declines substantially. Nonlinear least-squares regression involves iteratively adjusting these parameters—*y₀*, *x₀*, and *ω₀*—so that the RSS is minimized. This type of regression can be performed using various statistical tools, including Python. For example, in Python, the curve_fit function from the scipy.optimize module can be used to fit the model function. Nonlinear regression based on such model functions can help avoid overfitting, as observed in the polynomial regression shown in Figure 2. However, to further improve accuracy, incorporating weights based on sample size allows for fitting that reflects the reliability of the data.

1. **Weighted nonlinear least-squares regression**

When incorporating weights based on sample size, the RSS formula shown in Equation (1) is modified as shown in Equation (5).

$$\begin{aligned} \boldsymbol{RSS=}\sum_{\boldsymbol{i=1}}^{\boldsymbol{n}} {\boldsymbol{W}_{\boldsymbol{i}}\boldsymbol{\cdot}\left( \boldsymbol{Y}_{\boldsymbol{i}}\boldsymbol{-y}\left( \boldsymbol{x}_{\boldsymbol{i}} \right) \right)}^{\boldsymbol{2}}\boldsymbol{\#}\left( \boldsymbol{5} \right) \end{aligned}$$

Here, *W_i_* represents the weight assigned based on sample size. In this study, weights were calculated using the number of cases *N_i_* for each age group, with the formula *W_i_* = 1 / √*N_i_*. *N_i_* refers to the number of samples (cases) at each age. In Python, weighted nonlinear regression can be performed using the following syntax: curve_fit(model, Age, Live_Birth_Rate, p0=initial_parameter, sigma=Wi, absolute_sigma=True). In this example, “model” refers to a model function defined prior to calling curve_fit, with “Age” as the explanatory variable and “Live_Birth_Rate” as the outcome variable. The argument “p0” specifies the initial parameter values for the nonlinear regression. Care must be taken when setting these initial values, as solutions may fail to converge if the starting point is too far from the true parameters. If “sigma” and “absolute_sigma” are not specified, the function performs standard nonlinear regression without weighting. In contrast, specifying “sigma=Wi” and “absolute_sigma=True” enables weighted nonlinear least-squares regression based on *W_i_* = 1 / √*N_i_*. Figure 3 shows the results of applying both non-weighted and weighted nonlinear least-squares regression to the same dataset.


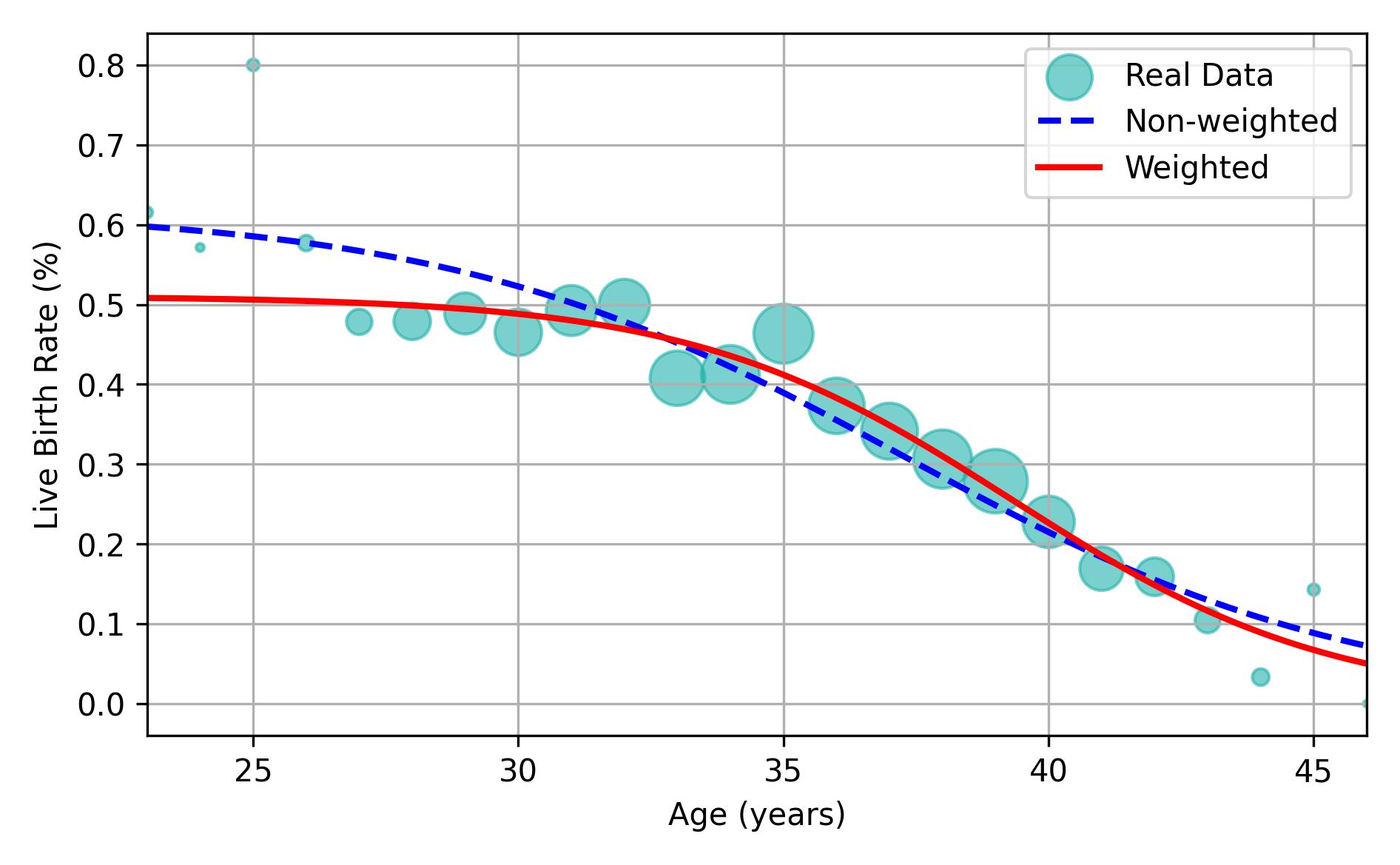


Figure 3. Comparison between weighted and unweighted nonlinear least-squares regression

“Real Data” represents the observed live birth rate at each age, with dot sizes scaled proportionally to the sample size to visually convey the variation in data volume across age groups. In age groups with smaller sample sizes—such as younger and older individuals—the observed values tend to exhibit greater fluctuation due to small-sample variation. The regression curve obtained from the non-weighted model is strongly influenced by outliers, such as those at ages 25 and 45, and deviates considerably from the observed values in the more data-rich age range of 35 to 40. In contrast, the weighted regression yields a smoother curve that closely follows the observed data in age groups with sufficient sample size, without being overly affected by outliers. These results suggest that weighted nonlinear least-squares regression is currently one of the most appropriate approximation methods, as it mitigates the effects of outliers and small-sample variability while producing estimates anchored in the most reliable portions of the data.
