# Supplementary material for: Comprehensive mathematical modeling of age-dependent oocyte quality and quantity for predicting live birth rate
Source: Front Endocrinol (Lausanne). 2025 Jun 9;16:1595970. doi: 10.3389/fendo.2025.1595970 (PMC12183067; doi:10.3389/fendo.2025.1595970)
Supplement: Supplementary file 1 [file DataSheet1.zip › Supplementary Materials/Supplementary Documents/Supplementary_document_2.docx]

Supplementary Document 2. Explanation of a logistic equation and its parameters

In this supplementary document, the logistic equation used as the model function is explained in detail. The logistic equation has long been utilized in biology and physiology as a mathematical model for population growth and decline. In reproductive medicine, data related to fertility decline frequently exhibit a declining logistic curve pattern. The fundamental form of the logistic equation is presented in Equation (1).

$$\begin{aligned} \boldsymbol{y}\left( \boldsymbol{x} \right)\boldsymbol{=}\frac{\boldsymbol{y}_{\boldsymbol{0}}}{\boldsymbol{1+}\mathbf{exp}\left( \frac{\boldsymbol{x-x}_{\boldsymbol{0}}}{\boldsymbol{\omega}_{\boldsymbol{0}}} \right)}\boldsymbol{\#}\left( \boldsymbol{1} \right) \end{aligned}$$

The explanatory variable *x* primarily represents female age in this study, whereas the dependent variable *y* corresponds to factors such as live birth rate, euploidy rate, and mean AMH level. The parameters optimized to minimize the sum of squared residuals between the actual data and the model function are *y_0_*, *x_0_*, and ω_0_.

1. y0: Maximum value (plateau) of the logistic curve

represents the highest value of the dependent variable and shares the same unit as the dependent variable.

1. x0: Half-peak age

shares the same unit as the explanatory variable and indicates the age at which the dependent variable declines to half of its maximum value (y0).

In this study, it is collectively referred to as “half-peak age”.

1. ω_0_: Decline width

shares the same unit as the explanatory variable and serves as a shape parameter that determines the slope of the decline curve. A smaller value results in a steeper decline, while a larger value leads to a more gradual decrease. In this study, it is collectively referred to as “decline width”.

Although curve fitting can be performed using the form of Equation 1, in this study, its fundamental form was converted into Equation 2 to provide more medically useful information.

$$\begin{aligned} \boldsymbol{y}\left( \boldsymbol{x} \right)\boldsymbol{=}\frac{\boldsymbol{y}_{\boldsymbol{0}}}{\boldsymbol{1+}\boldsymbol{3}^{\frac{\boldsymbol{x-}\boldsymbol{x}_{\boldsymbol{0}}}{\boldsymbol{\omega}}}}\boldsymbol{\#}\left( \boldsymbol{2} \right) \end{aligned}$$

This conversion enables the straightforward calculation of the ages at which the dependent variable declines to 90%, 75%, 50%, 25%, and 10% of its maximum value using the half-peak age (*x_0_*) and decline width (ω). This relationship, exemplified by the association between age and live birth rate, is summarized in Table 1 and Figure 1.

| **Table 1.** Description of the converted logistic equation parameters | | |
| --- | --- | --- |
| **Parameter** | **Name** | **Description** |
| *y_0_* | Max LBR | Maximum live birth rate |
| *x_0_* | Half-peak age | Age at which the live birth rate is half of the Max LBR |
| ω | Decline width | Defines the rate of LBR-declination within the interval of ω |
| *x_0_*–2ω | Age at 90% | Age at which the live birth rate is 90% of the Max LBR |
| *x_0_*–ω | Age at 75% | Age at which the live birth rate is 75% of the Max LBR |
| *x_0_*+ω | Age at 25% | Age at which the live birth rate is 25% of the Max LBR |
| *x_0_*+2ω | Age at 10% | Age at which the live birth rate is 10% of the Max LBR |
|  |  |  |


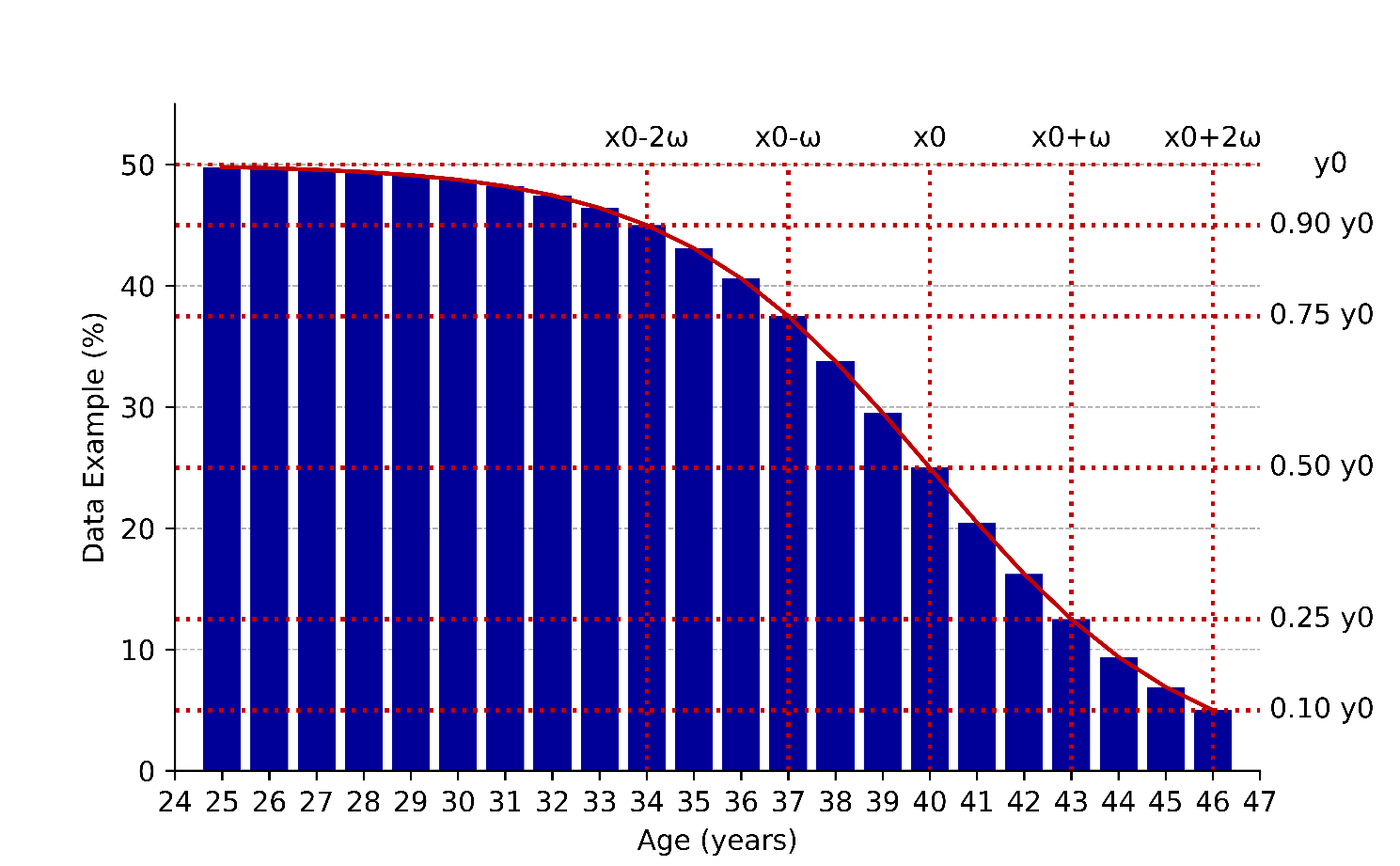


Figure 1. Graphical description of the converted logistic equation with the three parameters. This visually represents the curve fitting results using the three logistic equation parameters (*y0*, *x0*, and ω).

For example, as shown in Figure 1, if the half-peak age is 40 years and the decline width is 3 years, the ages at which the dependent variable declines to 90%, 75%, 50%, 25%, and 10% of its maximum value can be easily calculated as 34, 37, 40, 43, and 46 years, respectively, regardless of the maximum value.
