# Supplementary material for: Comprehensive mathematical modeling of age-dependent oocyte quality and quantity for predicting live birth rate
Source: Front Endocrinol (Lausanne). 2025 Jun 9;16:1595970. doi: 10.3389/fendo.2025.1595970 (PMC12183067; doi:10.3389/fendo.2025.1595970)
Supplement: Supplementary file 1 [file DataSheet1.zip › Supplementary Materials/Supplementary Documents/Supplementary_document_3.docx]

**Supplementary Document 3. Explanation of a gamma distribution function and its parameters**

In this supplementary document, we describe the details of a gamma distribution function used as the model function. In statistics, data distributions are typically represented using mean and standard deviation under the assumption of a normal distribution. However, in reproductive medicine, variables such as AMH, AFC, and number of mature oocytes per retrieval often exhibit asymmetric distributions that are skewed toward values close to zero. For such distributions, a gamma distribution function provides a more appropriate model. In a perfectly normal distribution, the mean, median, and mode are identical. However, in a gamma distribution, the general relationship is mean > median > mode. Therefore, representing data distributions solely with mean and standard deviation, assuming normality, fails to accurately reflect distribution characteristics. To properly represent the characteristics of asymmetric distributions like a gamma distribution, it is preferable to use additional measures of distribution spread, such as quartiles and percentiles, alongside mean and mode. A comparison between normal and gamma distributions is presented in Figure 1.


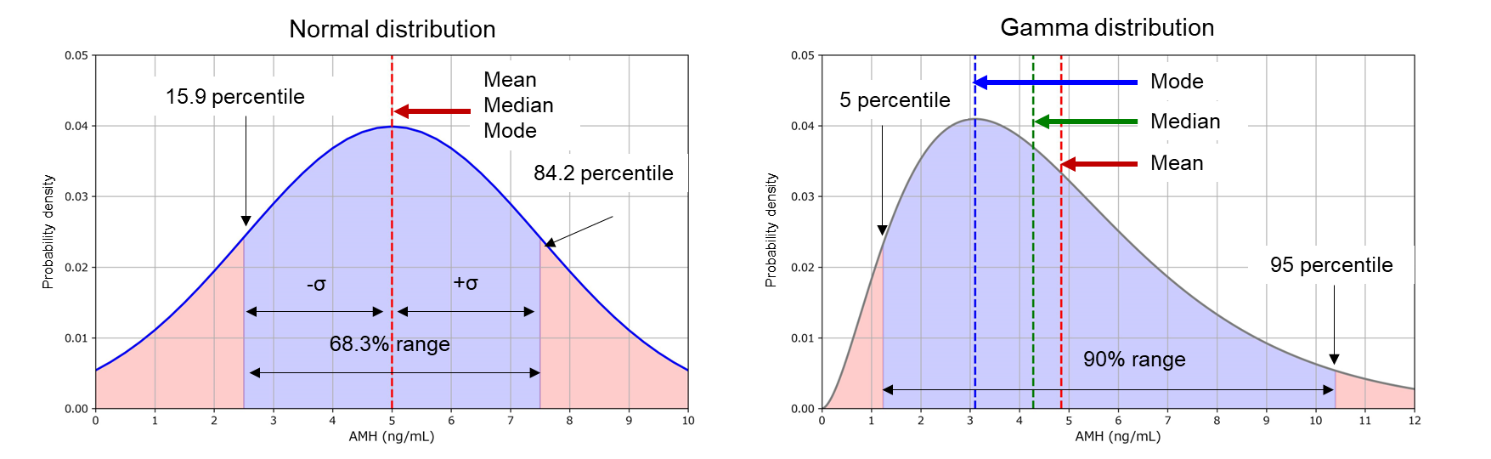


Figure 1. Comparison of normal distribution and gamma distribution. In a normal distribution, the mean, median, and mode are identical, whereas in a gamma distribution, the mode is positioned furthest to the left, followed by the median and then the mean, shifting progressively to the right. Additionally, in a gamma distribution, measures such as the 90% range (5th–95th percentiles) or interquartile range (25th–75th percentiles) would be more appropriate to represent the spread of the distribution. These characteristics distinguish the gamma distribution from the normal distribution, where standard deviation is typically used to indicate variability.

The fundamental form of the gamma distribution function is presented in Equation

$$\begin{aligned} \boldsymbol{y}\left( \boldsymbol{x} \right)\boldsymbol{=}\frac{\boldsymbol{x}^{\boldsymbol{\alpha-1}}\mathbf{exp}\left( \boldsymbol{-}\frac{\boldsymbol{x}}{\boldsymbol{\beta}} \right)}{\boldsymbol{\Gamma}\left( \boldsymbol{\alpha} \right)\boldsymbol{\beta}^{\boldsymbol{\alpha}}}\boldsymbol{\#}\left( \boldsymbol{1} \right) \end{aligned}$$

In this study, the explanatory variable x represents factors such as AMH levels, AFC, number of mature oocytes, and number of transferable embryos, whereas the dependent variable y represents the probability density of the distribution. The shape of this distribution is determined by two parameters: α (shape parameter) and β (scale parameter). These parameters are related to the mean and mode of the distribution. The relationships between the mean and mode are presented in Equations 2 and 3.

$$\begin{aligned} \boldsymbol{Mean=\alpha\cdot\beta\#}\left( \boldsymbol{2} \right) \end{aligned}$$

$$\begin{aligned} \boldsymbol{Mode=}\left( \boldsymbol{\alpha-1} \right)\boldsymbol{\cdot\beta\#}\left( \boldsymbol{3} \right) \end{aligned}$$

When age is a variable, the mean and mode can be approximated as described in Supplementary Document 1. Therefore, by constructing two-dimensional histogram data for each age group and performing two-dimensional weighted least-squares regression using the gamma distribution function to optimize the mean and mode, the overall distribution can be mathematically modeled. The formulas for calculating the two parameters of the gamma distribution from the mean and mode are presented in Equations 4 and 5.

$$\begin{aligned} \boldsymbol{\alpha=}\frac{\boldsymbol{Mean}}{\boldsymbol{Mean-Mode}}\boldsymbol{\#}\left( \boldsymbol{4} \right) \end{aligned}$$

$$\begin{aligned} \boldsymbol{\beta=Mean-Mode\#}\left( \boldsymbol{5} \right) \end{aligned}$$

Figure 2 presents the preliminary analysis graphs of curve fitting using the gamma distribution function for AMH histograms at ages 32, 37, and 42 years.


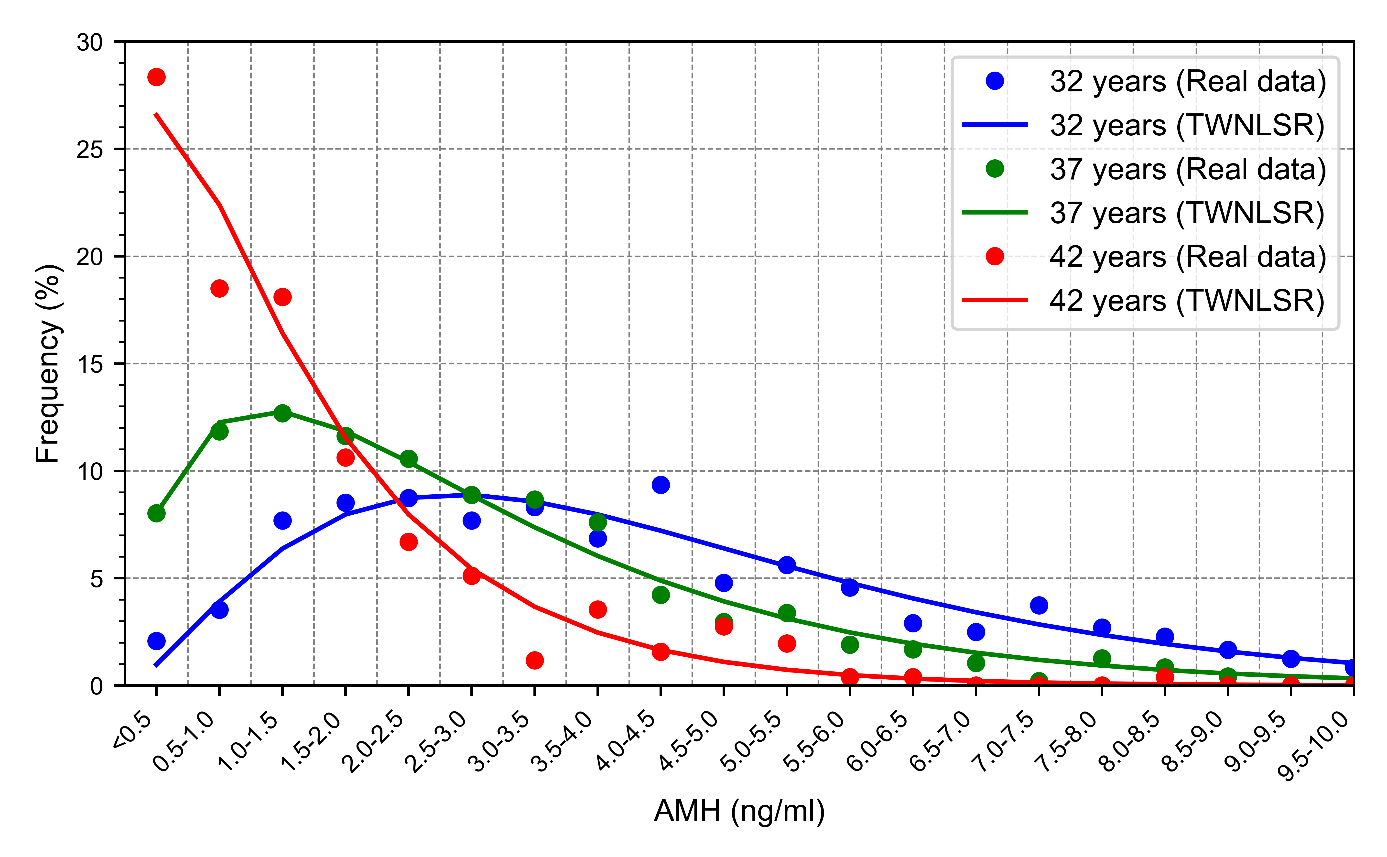


Figure 2. TWNLSR results of AMH distributions at ages 32, 37, and 42 years.

The gamma distribution function has the property that the probability density becomes zero when the explanatory variable is zero. However, AFC, number of mature oocytes, and number of transferable embryos can take a value of zero. In particular, the number of transferable embryos is frequently zero in patients of advanced age. Therefore, when performing curve fitting using the gamma distribution function, it is necessary to offset the explanatory variable by +1. When calculating the mean and mode after curve fitting, a correction of -1 is required to return to the original scale. In the calculation of gamma distribution parameters for AFC, number of mature oocytes, and number of transferable embryos (Equations 4 and 5), the mean and mode are computed with an offset of +1, and the explanatory variable is also processed with +1 when computing the probability density. On the other hand, for graph labeling, the values should be displayed on the original scale.
