# Supplementary material for: Comprehensive mathematical modeling of age-dependent oocyte quality and quantity for predicting live birth rate
Source: Front Endocrinol (Lausanne). 2025 Jun 9;16:1595970. doi: 10.3389/fendo.2025.1595970 (PMC12183067; doi:10.3389/fendo.2025.1595970)
Supplement: Supplementary file 1 [file DataSheet1.zip › Supplementary Materials/Supplementary Documents/Supplementary_document_4.docx]

**Supplementary Document 4. Explanation of the cumulative probability function and exponential decay**

In this supplementary document, we describe the details of the cumulative probability function and exponential decay, which were used as model functions in the section on live birth rate per OPU.

1. Cumulative Probability Function

The relationship between quantifying factors and live birth rate exhibited a cumulative probability function, which is calculated by Equation 1.

$$\begin{aligned} \boldsymbol{y}\left( \boldsymbol{x} \right)\boldsymbol{=1-}\left( \boldsymbol{1-p} \right)^{\boldsymbol{x}}\boldsymbol{\#}\left( \boldsymbol{1} \right) \end{aligned}$$

In this study, the explanatory variable *x* represents factors such as AMH, AFC, number of mature oocytes, and number of transferable embryos, whereas the dependent variable *y* represents the live birth rate per oocyte retrieval. The shape of this distribution is determined by a single parameter, the probability coefficient *p*.

The reason this formula applies can be easily understood by considering transferable embryos as an example. The probability coefficient *p* represents the average live birth rate per transferable embryo. Since embryo transfer can be repeated as many times as the number of transferable embryos, this formula holds. Therefore, the probability coefficient *p* indicates the average live birth rate per unit of each quantifying factor.

Figure 1 illustrates the relationship between each quantifying factor and the live birth rate per OPU comparing the actual data (dots) with the curve fitting results obtained using WNLSR based on Equation 1 (curve).


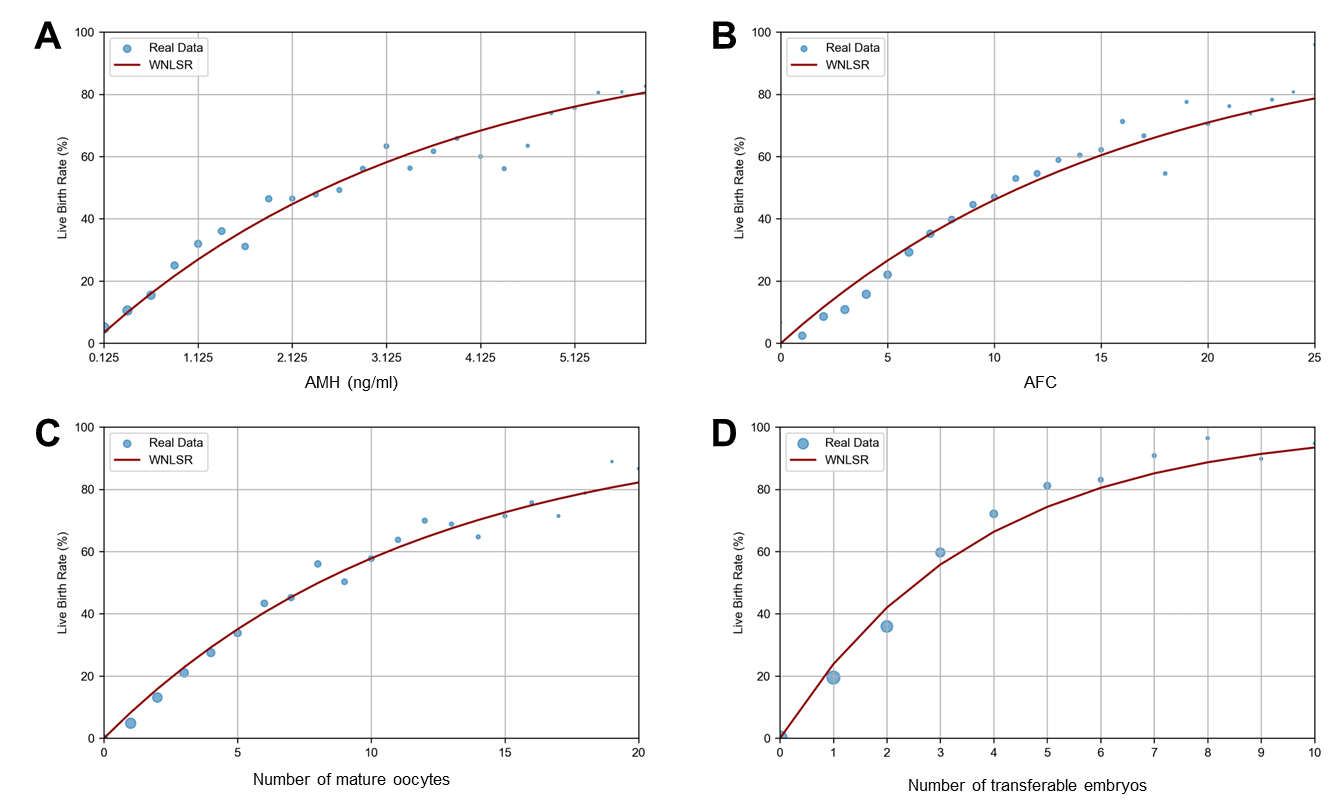


Figure 1. WNLSR results of quantifying factors versus live birth rates per OPU. (A) AMH, (B) AFC, (C) number of mature oocytes, (D) number of transferable embryos.

2. Exponential decay

The fundamental formula for exponential decay is presented in Equation 2.

$$\begin{aligned} \boldsymbol{y}\left( \boldsymbol{x} \right)\boldsymbol{=}\boldsymbol{y}_{\boldsymbol{0}}\boldsymbol{\cdot}\mathbf{exp}\left( \boldsymbol{-}\frac{\boldsymbol{x-1}}{\boldsymbol{\omega}_{\boldsymbol{0}}} \right)\boldsymbol{\#}\left( \boldsymbol{2} \right) \end{aligned}$$

In this study, the explanatory variable *x* represents the number of retrieval cycles, while the dependent variable *y* represents the live birth rate per oocyte retrieval. The initial value *y_0_* indicates the live birth rate at the first OPU, and the decay parameter ω_0_ represents the rate of decline, with smaller values indicating a more rapid decline and larger values indicating a more gradual decline. To ensure that each parameter provides meaningful clinical information, Equation 2 was transformed into the form of Equation 3 for analysis in this study.

$$\begin{aligned} \boldsymbol{y}\left( \boldsymbol{x} \right)\boldsymbol{=}\boldsymbol{y}_{\boldsymbol{0}}\boldsymbol{\cdot}\mathbf{2}^{\left( \frac{\boldsymbol{x-1}}{\boldsymbol{\omega}} \right)}\boldsymbol{\#}\left( \boldsymbol{3} \right) \end{aligned}$$

Through this transformation, it can be calculated that if OPU is repeated ω times from the first retrieval, the live birth rate decreases to 50% of its initial value, and if retrieval is repeated 2ω times, it declines to 25% of the initial value.

However, in univariate analysis, it is necessary to account for the effects of confounding factors. In practice, patients with advanced age and/or lower AMH levels tend to repeat oocyte retrieval cycles, suggesting that these factors may contribute to the exponential decline in the live birth rate. Therefore, performing logistic regression analysis by incorporating age and quantifying factors as explanatory variables is crucial to adjust for the actual effect of the number of retrieval cycles on the live birth rate and to estimate the adjusted odds ratio (aOR).
